# Measurement of Respiratory Chain Enzyme Activity in Human Renal Biopsy Specimens

**DOI:** 10.3390/jcm6090090

**Published:** 2017-09-19

**Authors:** Arun Ghose, Christopher M. Taylor, Alexander J. Howie, Anapurna Chalasani, Iain Hargreaves, David V. Milford

**Affiliations:** 1 Department of Nephrology, Birmingham Children’s Hospital, Birmingham B4 6NH, UK; arun.ghose@bch.nhs.uk (A.G.); cmarktaylor@hotmail.com (C.M.T.); 2 Department of Histopathology, Birmingham Children’s Hospital, Birmingham B4 6NH, UK; alexander.howie@nhs.net; 3 Neurometabolic Unit, National Hospital for Neurology and Neurosurgery, London WC1N 3BG, UK; annapurnachalasani@nhs.net (A.C.); i.hargreaves@ucl.ac.uk (I.H.)

**Keywords:** mitochondrial disorders, renal biopsy, respiratory chain enzymes

## Abstract

*Background*: Mitochondrial disorders can present as kidney disease in children and be difficult to diagnose. Measurement of mitochondrial function in kidney tissue may help diagnosis. This study was to assess the feasibility of obtaining renal samples and analysing them for respiratory chain enzyme activity. *Methods*: The subjects were children undergoing a routine diagnostic renal biopsy, in whom a clinical condition of renal inflammation, scarring and primary metabolic disorder was unlikely. A fresh sample of kidney was snap frozen and later assayed for the activities of respiratory chain enzyme complexes I, II/III, and IV using spectrophotometric enzyme assay, and expressed as a ratio of citrate synthase activity. *Results*: The range of respiratory chain enzyme activity for complex I was 0.161 to 0.866 (mean 0.404, SD 0.2), for complex II/III was 0.021 to 0.318 (mean 0.177, SD 0.095) and for complex IV was 0.001 to 0.025 (mean 0.015, SD 0.006). There were correlations between the different activities but not between them and the age of the children or a measure of the amount of chronic damage in the kidneys. *Conclusion*: It is feasible to measure respiratory chain enzyme activity in routine renal biopsy specimens.

## 1. Introduction

Mitochondrial disorders are difficult to diagnose clinically because of phenotypic variation. Renal presentations include the renal Fanconi syndrome (tubular wasting of bicarbonate, sodium and potassium, glucose and amino acids) and steroid unresponsive nephrotic syndrome as a result of glomerular damage; progression to chronic kidney disease is also described [1,2,3]. Mitochondrial cytopathy is important to diagnose, as there may be reversibility of those with coenzyme Q10 biosynthesis defects as well as the implication for treatment options, including renal transplantation. A clue to the diagnosis may come from clinical abnormalities of other organs that require a high rate of oxidative metabolism, such as the brain (encephalopathy, retinopathy or stroke), skeletal muscle (myopathy), heart (cardiomyopathy) and liver; a full review of the clinical features of mitochondrial diseases is beyond the scope of this paper, but is extensively reviewed by Gorman et al. [4]. Without these other features, diagnosis may be difficult in children who have a predominantly renal presentation. Also, the proportion of normal to abnormal mitochondria may vary in different organs (heteroplasmy). Consequently, a diagnostic procedure performed on skeletal muscle, for example, may not identify an abnormality in a child whose main organ involvement is kidney or brain. A logical approach is to investigate tissue from the affected organ if possible [5,6,7,8].

Renal biopsy is a routine diagnostic procedure in children, principally to provide material for histopathological examination, but this can also be a source of appropriate tissue for enzymatic studies. Because mitochondrial disorders are likely to affect respiratory chain enzyme activity (RCEA), assay of this activity in renal biopsies could facilitate the diagnosis of these disorders. Before the measurement of RCEA could be investigated for its value in the diagnosis of mitochondrial disorders, it was necessary to see whether RCEA could be assayed in human renal tissue obtained by routine renal biopsy. The aim of this study was to determine the feasibility of measuring RCEA in renal biopsy specimens from children. The results of this study will provide pilot data for a more thorough validation study to assess the reliability of renal RCEA assessment as a diagnostic investigation.

## 2. Experimental Section

### 2.1. Renal Biopsies

Research and ethical approval was gained from the North Staffordshire Local Research Ethics Committee prior to the study (REC reference number: 08/H1204/123). Information for parents and information appropriate for the age of children was provided. Written parental consent was obtained, as was written consent from children over 14 years old.

Children aged from three to 16 years of age undergoing routine renal biopsy were invited to participate in the study. Samples were obtained using a 14-gauge biopsy needle to give two cores, and these were inspected immediately with a dissecting microscope. If there were more than 18 mm of cortex, a 3-mm piece of cortex was snap frozen in liquid nitrogen. In biopsies without 18 mm of cortex, and if there were more than 15 mm of medulla, a 3 mm core of medulla was taken. Only one research sample was taken from each patient. The remaining tissue was processed routinely for diagnosis. The frozen sample was only released for analysis of RCEA once the pathologist’s report confirmed no more than minor structural abnormalities. If the diagnosis required more tissue, the frozen sample was formalin fixed for routine processing.

Biopsies from 13 children were considered suitable for RCEA assay. A sample from another child was not used as there was insufficient material for routine diagnostic studies. All these children had two kidneys of normal size, normal estimated glomerular filtration rate, and normal blood pressure. The indication for biopsy was persistent haematuria in six, nephrotic syndrome in four, persistent proteinuria in one, and treatment of the nephrotic syndrome with a calcineurin inhibitor, to check if chronic renal damage had resulted, in two. No child had any abnormalities outside the kidney to suggest a mitochondrial disorder.

Diagnoses were made in orthodox ways, and are given in the Results. Because some specimens had microscopically visible chronic damage, seen as tubular atrophy and/or global sclerosis of glomeruli, a morphometric method was used to measure this. Areas of chronic damage in the cortex were outlined on computer images of sections stained by periodic acid-methenamine silver, expressed as a percentage of the whole cortical cross-sectional area, and called the index of chronic damage [9].

### 2.2. Tissue Homogenization

Samples were immediately put on dry ice and then stored at −70 °C. Before RCEA assay, samples were homogenized on ice, 1:9 (*w*/*v*), in 320 mmol/L sucrose, 1 mmol/L ethylenediamine tetra acetic acid dipotassium salt, and 10 mmol/L Trizma-base, pH 7.4, using a pre-chilled hand-held glass homogenizer. Approximately 10–15 mg of renal tissue was homogenized per sample, distributed into four Eppendorf tubes. One Eppendorf tube was used for each enzyme determination following three cycles of freeze/thawing [7].

### 2.3. RCEA Assays

Mitochondrial respiratory chain enzyme and citrate synthase (CS) activities were determined by spectrophotometric enzyme assay [8,10]. Complex I (NADH:ubiquinone reductase, EC 1.6.5.3) activity was measured by the rotenone sensitive oxidation of NADH at 340 nm. Complex II/III (succinate:cytochrome c reductase, EC 1.3.5.1 and EC 1.10.2.2) activity was measured by antimycin A-sensitive succinate dependent reduction of cytochrome c at 550 nm. Complex IV (cytochrome c oxidase, EC 1.9.3.1) activity was measured by the potassium cyanide sensitive oxidation of reduced cytochrome c at 550 nm. CS (EC 2.3.3.1) activity was determined by the formation of 5-thio-2-nitrobenzoic acid following the incubation of tissue homogenate with acetyl-CoA, oxaloacetate and 5,5′-dithiobis-(2-nitrobenzoic acid), at 412 nm. No reference wavelength was used in the enzyme assays.

All mitochondrial RCEAs were expressed as a ratio to the activity of CS, a mitochondrial marker enzyme, to standardise the mitochondrial enrichment of the sample [11]. Because activities of CS and complexes I and II/III were expressed as nmol/min/mL, but activity of complex IV was expressed as k/min/mL, the ratios of complex I and complex II/III activities to CS activity have no units, but the ratio of complex IV activity to CS activity has units of k/nmol. The range, mean and standard deviation were calculated for each RCEA. Correlations between age, index of chronic damage and each RCEA were determined by Spearman’s rank correlation coefficient (*r*), and significant correlations were considered to be those with the conventional *p* value of under 0.05.

## 3. Results

Patient details, diagnoses, indexes of chronic damage, measurements of RCEA, and whether measurements were on cortex or medulla or on undetermined renal tissue are given in Table 1.

The range of RCEA for renal complex I was 0.161 to 0.866 (mean 0.404, SD 0.2), for complexes II/III was 0.021 to 0.318 (mean 0.177, SD 0.095) and for complex IV was 0.001 to 0.025 (mean 0.015, SD 0.006). There were no significant correlations between age and index of chronic damage, age and any RCEA, or index of chronic damage and any RCEA. RCEA of complex I showed a trend to correlate with RCEA of complexes II/III (*r* = 0.539, *p* = 0.057) and was significantly correlated with RCEA of complex IV (*r* = 0.704, *p* = 0.007). RCEA of complexes II/III was significantly correlated with RCEA of complex IV (*r* = 0.660, *p* = 0.014). The inter-assay coefficient of variation values for the RCEA and CS enzyme measurements calculated from the in-house QC human skeletal muscle homogenate data are as follows: Complex I: 9.11% (*n* = 29); Complex II/III: 10.70% (*n* = 29); Complex IV: 8.9% (*n* = 29); CS: 5.6% (*n* = 29). QC muscle samples were prepared from skeletal muscle tissue from patients with no biochemical evidence of a RCEA defect by the same method as the kidney homogenates were prepared.

## 4. Discussion

The DNA coding for mitochondrial respiratory chain enzymes NADH-Q oxidoreductase (complex I), succinate-Q reductase (complex II), Q-cytochrome c oxidoreductase (complex III) and complex IV (cytochrome c oxidase) is a mixture of nuclear genes and mitochondrial genes. Most disorders of RCEA in children are caused by mutations in nuclear genes, although mutations in mitochondrial DNA have also been found [13]. In some cases, genetic screening does not give a diagnosis, and so further investigations may be required. Another problem with mitochondrial respiratory chain disorders is that mutations may only be expressed in affected tissues. Consequently, assessment of the affected organ may be appropriate. Confirmation of abnormal RCEA may then be helpful in determining the most appropriate genetic tests. This study has indicated that it is possible to measure RCEA in kidney tissue obtained by the routine clinical procedure of renal biopsy.

Using CS activity as a reference controlled for variables such as the mitochondrial enrichment of the sample [11]. There was a wide range of RCEA for each of the complexes, with a 5-fold difference between the minimum value and maximum value for complex I, a 15-fold difference for complex II/III, and a 25-fold difference for complex IV (Table 1), although RCEA of the different complexes had significant or nearly significant correlations between each other. The mean RCEA of complex I was higher than the ranges for skeletal muscle and liver, the mean RCEA of complex II/II was towards the upper end of the ranges for skeletal muscle and liver, and the mean RCEA of complex IV was towards the lower end of the ranges for skeletal muscle and liver, although the RCEA in kidney were derived from a small sample. We cannot also exclude the possibility that poor sample handling of some of the renal biopsies may have contributed to loss of RCEA and therefore may account for the wide range of enzyme activities [14].

This study has a number of limitations. The number of patients sampled was small and most children had patchy chronic damage as assessed by the index of chronic damage measured in the histological specimen. However, as there was no way of knowing if the sample used for RCEA contained areas of chronic damage and the percentage of the biopsy specimen affected by chronic change was generally low (greater than 5% in only one child) it is not unreasonable to consider the values obtained as being representative of activity in normal renal tissue. Unfortunately, the small sample size precluded repeat analyses to confirm reproducibility, and so could explain the range of values obtained. Furthermore, no child with known mitochondrial disease presented during the time of this study to allow comparison with the values we obtained. Some workers have proposed validating the methodology by undertaking RCEA studies in nephrectomy samples, as these would provide a larger amount of renal tissue and allow reproducibility studies. However, we believe these samples would be unrepresentative; firstly, because the tissue would be exposed to significant ischaemia following clamping of the renal artery prior to the nephrectomy; and secondly, because the nephrectomy specimen would predominantly comprise diseased tissue. Importantly, given the heterogeneity of mitochondrial respiratory chain disorders, a reference range derived from true ethical controls may be required to accurately determine RCEA dysfunction in human renal tissue.

We have shown no relationship between the age of the children and RCEA in renal tissue when enzyme activities are expressed as a ratio to CS. Although this has previously been reported for RCEA in muscle and liver [12], this is the first time it has been reported for renal tissue. This is important since it indicates that age-specific reference intervals may not be necessary for renal RCEA if enzyme activities are expressed as a ratio to CS activity. The site of origin of the tissue analysed, whether cortex or medulla, also appeared unimportant. However, at present it cannot be excluded that there may be a difference in RCEA between these two regions of the kidney and future studies to confirm or refute this will be undertaken.

The lack of a relation between the index of chronic damage and RCEA is encouraging for future studies of RCEA, because the measured activities presumably reflect those in surviving tubules, suggesting that assays can still be applied to samples from children with a suspected mitochondrial disorder but with chronic kidney disease.

We have shown that it is feasible to obtain samples from routine renal biopsies and freeze them for later analysis of RCEA. However, as there was insufficient tissue to duplicate analysis, the effect of poor sample handling, together with uncertainty about whether tissue from the renal medulla or cortex have different RCEA, this study is unable to establish a RCEA reference interval for renal tissue. It will require a larger and more rigorous validation study using ethically obtained control tissue, as well as renal biopsies from patients with confirmed mitochondrial respiratory chain dysfunction, to establish a reference range and to confirm the diagnostic value of this test. It remains to be seen if such a study is possible, given the difficulty in obtaining sufficient tissue from human renal biopsy.

## Figures and Tables

**Table 1:** Clinical details, index of chronic damage, and assay of respiratory chain enzyme activities (RCEA) in cortex or medulla, if site of tissue known. RCEA are expressed as a ratio of citrate synthase activity. Normal RCEA for skeletal muscle and liver are included for comparison [8].

Sex, Age (year)	Diagnosis	Index of Chronic Damage	Complex I	Complex II/III	Complex IV (k/nmol)	Cortexor Medulla or Not Known
M3	minimal change	0%	0.176	0.021	0.003	Cortex
M7	minimal change	3%	0.161	0.166	0.015	Cortex
M9	CNI effects	4%	0.542	0.042	0.018	Cortex
M10	Alport syndrome	2%	0.522	0.253	0.025	Cortex
F11	IgA nephropathy	3%	0.220	0.120	0.016	Medulla
F13	IgA nephropathy	3%	0.229	0.105	0.001	cortex
M13	CNI effects	15%	0.388	0.238	0.017	Cortex
F14	thin gbm disease	3%	0.866	0.313	0.018	not known
F14	IgA nephropathy	5%	0.569	0.225	0.016	not known
M15	thin gbm disease	0%	0.516	0.224	0.018	Cortex
F15	seg. sclerosis	2%	0.333	0.105	0.014	Medulla
F16	no abnormality	0%	0.452	0.318	0.018	Cortex
M16	minimal change	0%	0.278	0.172	0.017	Cortex
normal RCEA values for skeletal muscle and liver						
	skeletal muscle		0.104–0.268	0.040–0.204	0.014–0.034	
	liver		0.054–0.22	0.057–0.204	0.011–0.031 Reference ranges for RCEA in liver and muscle are not thought to be age dependent according to the study of Chretien et al. [12]	

Abbreviations: CNI, calcineurin inhibitor; gbm, glomerular basement membrane; seg, segmental.
